# Supplementary material for: Chemical Constituents, Antioxidant Potential, and Antimicrobial Efficacy of Pimpinella anisum Extracts against Multidrug-Resistant Bacteria
Source: Microorganisms. 2023 Apr 14;11(4):1024. doi: 10.3390/microorganisms11041024 (PMC10144661; doi:10.3390/microorganisms11041024)
Supplement: Supplementary file 1 [file microorganisms-11-01024-s001.zip › microorganisms-2304319-supplementary.pdf]

Table S1. Results of the VITEK2 biochemical testing for *Enterococcus faecalis*

2	APPA	+	3	ADO	-	4	PyrA	-	5	IARL	-	7	dCEL	-	9	BGAL	+
10	H2S	+	11	BNAG	-	12	AGLTP	-	13	dGLU	+	14	GGT	-	15	OFF	+
17	BGLU	-	18	dMAL	+	19	dMNE	-	20	dMNE	+	21	BXYL	-	22	BAlaP	-
23	PrOA	+	26	LIP	-	27	PLE	-	29	TYrA	+	31	URE	-	32	dSOR	+
33	SAC	+	34	dTAG	-	35	dTRE	+	36	CIT	-	37	MNT	-	39	5KG	-
40	ILATK	+	41	AGLU	-	42	SUCT	+	43	NAGA	-	44	AGAL	-	45	PHOS	-
45	GLyA	+	47	ODC	+	48	LDC	+	53	IHISa	-	56	CMT	+	57	BGUR	-
58	O129R	+	59	GGAA	-	61	ImLTa	+	62	ELLM	-	64	ILARA	+			

Results of the VITEK2 biochemical testing for *Staphylococcus aureus*

2	APPA	-	3	ADO	-	4	PyrA	-	5	IARL	-	7	dCEL	-	9	BGAL	+
10	H2S	+	11	BNAG	-	12	AGLTP	-	13	dGLU	+	14	GGT	-	15	OFF	+
17	BGLU	+	18	dMAL	+	19	dMNE	+	20	dMNE	+	21	BXYL	-	22	BAlaP	-
23	PrOA	-	26	LIP	-	27	PLE	-	29	TYrA	+	31	URE	-	32	dSOR	+
33	SAC	+	34	dTAG	-	35	dTRE	+	36	CIT	-	37	MNT	-	39	5KG	+
40	ILATK	+	41	AGLU	+	42	SUCT	+	43	NAGA	-	44	AGAL	+	45	PHOS	-
45	GLyA	-	47	ODC	+	48	LDC	+	53	IHISa	-	56	CMT	+	57	BGUR	-)
58	O129R	+	59	GGAA	-	61	ImLTa	-	62	ELLM	+	64	ILARA	-			

Results of the VITEK2 biochemical testing for *Salmonella typhi*

2	APPA	+	3	ADO	-	4	PyrA	-	5	IARL	-	7	dCEL	-	9	BGAL	+
10	H2S	-	11	BNAG	-	12	AGLTP	-	13	dGLU	+	14	GGT	-	15	OFF	+
17	BGLU	+	18	dMAL	+	19	dMNE	+	20	dMNE	+	21	BXYL	-	22	BAlaP	-
23	PrOA	-	26	LIP	-	27	PLE	+	29	TYrA	+	31	URE	-	32	dSOR	+
33	SAC	+	34	dTAG	-	35	dTRE	+	36	CIT	-	37	MNT	-	39	5KG	-
40	ILATK	+	41	AGLU	-	42	SUCT	+	43	NAGA	-	44	AGAL	+	45	PHOS	-
45	GLyA	-	47	ODC	+	48	LDC	+	53	IHISa	-	56	CMT	+	57	BGUR	-
58	O129R	+	59	GGAA	-	61	ImLTa	-	62	ELLM	+	64	ILARA	+			

Results of the VITEK2 biochemical testing for *Pseudomonas aeruginosa*

2	APPA	-	3	ADO	-	4	PyrA	-	5	IARL	-	7	dCEL	-	9	BGAL	+
10	H2S	-	11	BNAG	-	12	AGLTP	-	13	dGLU	+	14	GGT	-	15	OFF	+
17	BGLU	-	18	dMAL	+	19	dMNE	+	20	dMNE	+	21	BXYL	+	22	BAlaP	-
23	PrOA	+	26	LIP	-	27	PLE	-	29	TYrA	+	31	URE	-	32	dSOR	+
33	SAC	-	34	dTAG	-	35	dTRE	+	36	CIT	-	37	MNT	-	39	5KG	-
40	ILATK	+	41	AGLU	-	42	SUCT	+	43	NAGA	-	44	AGAL	+	45	PHOS	-
45	GLyA	-	47	ODC	+	48	LDC	+	53	IHISa	-	56	CMT	+	57	BGUR	+
58	O129R	+	59	GGAA	-	61	ImLTa	-	62	ELLM	+	64	ILARA	-			

Results of the VITEK2 biochemical testing for *Acinetobacter baumannii*

2	APPA	-	3	ADO	-	4	PyrA	-	5	IARL	-	7	dCEL	-	9	BGAL	+
10	H2S	+	11	BNAG	-	12	AGLTP	-	13	dGLU	+	14	GGT	-	15	OFF	+
17	BGLU	+	18	dMAL	+	19	dMNE	+	20	dMNE	+	21	BXYL	+	22	BAlaP	-
23	PrOA	+	26	LIP	-	27	PLE	-	29	TYrA	+	31	URE	_	32	dSOR	+
33	SAC	+	34	dTAG	-	35	dTRE	+	36	CIT	-	37	MNT	-	39	5KG	-
40	ILATK	+	41	AGLU	-	42	SUCT	+	43	NAGA	-	44	AGAL	+	45	PHOS	-
45	GLyA	-	47	ODC	+	48	LDC	+	53	IHISa	-	56	CMT	+	57	BGUR	-
58	O129R	+	59	GGAA	-	61	ImLTa	+	62	ELLM	+	64	ILARA	-			
